# Biochemical, Immunohistochemical and Behavioral Effects of Spexin in a Methimazole-Induced Hypothyroidism Rat Model

**DOI:** 10.3390/biology15120932

**Published:** 2026-06-15

**Authors:** Seda Koçak, Gülhan Ünlü, Kübra Tuğçe Kalkan, Ferhat Pektaş, Ahmet Türk

**Affiliations:** 1 Department of Physiology, Faculty of Medicine, Kırşehir Ahi Evran University, Kırşehir 40100, Türkiye; 2 Department of Pharmacology, Faculty of Medicine, Kırşehir Ahi Evran University, Kırşehir 40100, Türkiye; 3 Department of Embryology and Histology, Faculty of Medicine, Kırşehir Ahi Evran University, Kırşehir 40100, Türkiye; 4 Department of Embryology and Histology, Faculty of Medicine, Adıyaman University, Kırşehir 02040, Türkiye

**Keywords:** behavior, hypothyroid, irisin, kisspeptin, spexin

## Abstract

Hypothyroidism is associated with metabolic, neurobehavioral, and reproductive alterations that may involve neuroendocrine regulatory peptides. Spexin, a neuropeptide implicated in energy homeostasis, has recently attracted attention for its possible role in thyroid and reproductive axis regulation. Therefore, this study aimed to investigate the effects of spexin on neurobehavioral responses and the tissue-specific expression of irisin and KISS-1 in the cerebral cortex and testis under hypothyroid conditions. Thirty-two male *Wistar albino* rats were randomly divided into four groups: Control, Hypothyroid (methimazole, 0.03% in drinking water for 35 days), Hypothyroid + Spexin (methimazole plus spexin, 25 µg/kg, intraperitoneally), and Spexin (25 µg/kg, intraperitoneally). Behavioral assessments were performed using the Open Field Test and Forced Swim Test. Serum thyroid hormone levels were analyzed, and brain and testis tissues were evaluated immunohistochemically for irisin and KISS-1 expression. Hypothyroid rats showed increased thyroid-stimulating hormone levels, decreased thyroxine concentrations. Spexin administration significantly reduced TSH levels and increased T4 concentrations. Spexin treatment reduced thigmotaxis compared to controls. No significant differences were found among groups in overall locomotor activity, time spent in the central zone, or FST parameters. Immunohistochemical analyses demonstrated reduced irisin and KISS-1 expression in hypothyroid rats, which was restored following spexin treatment. In conclusion, spexin exerted TSH-suppressive and T4-enhancing effects in experimental hypothyroidism. Its effects on irisin and KISS-1 expression suggest potential involvement in neuroendocrine and reproductive axis regulation.

## Simple Summary

Hypothyroidism is a common endocrine disorder characterized by insufficient thyroid hormone production and is associated with reproductive and biomarker-related disturbances. Thyroid hormone deficiency is linked with depression, anxiety, reduced activity, and measurable cognitive slowing and memory problems. Spexin, a neuropeptide associated with energy metabolism, neuroendocrine and reproductive processes, has been suggested to play a role in hypothalamic–pituitary signaling and thyroid hormone regulation. Therefore, this study aimed to investigate the effects of spexin administration on the simultaneous tissue expressions of irisin and KISS-1 in both the cerebral cortex and testis, while evaluating the associated biochemical, neurobehavioral responses in a rat model of methimazole-induced hypothyroidism.

## 1. Introduction

Hypothyroidism, one of the most common endocrine disorders, is characterized by a deficiency of thyroid hormones and is typically defined by alterations in thyroid-stimulating hormone (TSH) and free thyroxine (fT4) levels. Although numerous studies have investigated the prevalence of hypothyroidism, reported rates vary depending on whether individuals have been diagnosed and treated or remain undiagnosed [1,2,3].

In experimental animal studies, hypothyroidism is commonly induced using methimazole, which inhibits the thyroid peroxidase enzyme and thereby suppresses thyroid hormone synthesis. Administration of specific doses of methimazole in rats leads to decreased serum triiodothyronine (T3) and thyroxine (T4) levels, accompanied by an increase in TSH levels [4]. The close similarity of these histological features to those observed in human hypothyroidism further supports the clinical relevance of this model [5]. Thyroid problems can cause changes in hormone levels, which in turn may lead to anxiety and depression-like behaviors. These issues can also affect motor skills, learning, and memory. These effects are important for understanding how thyroid dysfunction can impact mental health and brain functions [6,7]. These findings indicate that experimentally induced hypothyroidism is associated not only with endocrine changes but also with significant neuropsychological effects.

The hypothalamus secretes gonadotropin-releasing hormone (GnRH), which stimulates the anterior pituitary gland to release follicle-stimulating hormone (FSH) and luteinizing hormone (LH), thereby regulating spermatogenesis and testosterone production in males [8]. The testis was long considered an organ unresponsive to thyroid hormones; however, increasing evidence demonstrating the presence of functional thyroid hormone receptors in testicular tissue has challenged this view. Recent findings suggest that thyroid hormones play an important role in testicular function [9]. In particular, triiodothyronine (T3) has been shown to regulate Sertoli cell proliferation and differentiation during testicular development, including the formation of the blood–testis barrier [10]. These observations indicate that thyroid hormones exert regulatory effects on the hypothalamic–pituitary–gonadal (HPG) axis.

Spexin is a neuropeptide belonging to the galanin family and consists of a 14-amino acid sequence. It is secreted by hypothalamic neurons as well as by various peripheral tissues and is involved in energy metabolism, endocrine regulation, and reproductive functions [11]. Altered spexin levels have been reported in disorders related to energy homeostasis and reproduction, including obesity, diabetes, and polycystic ovary syndrome. Furthermore, neurons responsible for GnRH synthesis appear to be directly sensitive to spexin [12,13]. Spexin mainly signals through GALR2/3-coupled PKC/PKA/MAPK/AKT/STAT3 pathways to modulate GnRH neurons, pituitary gonadotropes, and gonadal cells [14,15,16,17]. This neuropeptide can link metabolic–reproductive roles within the HPG axis represents coordinating energy homeostasis with fertility [15].

Kisspeptin, another bioactive peptide related to spexin, plays a key role in metabolic, reproductive, and behavioral processes [11]. Irisin, a myokine/adipokine, is associated with metabolic diseases, and previous studies have shown that kisspeptin-10 (KISS-10) can increase irisin secretion, potentially via neuropeptide Y (NPY)-mediated pathways [18]. In addition, irisin has been reported to induce early puberty by dose-dependently increasing levels of GnRH, kisspeptin, neurokinin B (NKB), LH, FSH, and estradiol [19]. Kisspeptin exerts its effects by binding to its receptor, kisspeptin receptor (KISS1R), thereby activating signaling pathways that stimulate GnRH secretion and regulate the HPG axis [20]. Collectively, current evidence suggests that spexin, irisin, and kisspeptin may be involved in HPG axis regulation in hypothyroidism, a condition closely associated with hormonal imbalance and reproductive dysfunction.

The present study aims to investigate the effects of spexin on thyroid dysfunction in methimazole-induced hypothyroid rats. In addition, immunohistochemical changes in irisin and kisspeptin expression in brain and testicular tissues, as well as alterations in behavioral parameters associated with thyroid hormone interactions, were evaluated.

## 2. Materials and Methods

### 2.1. Experimental Groups

In our study, 32 male Wistar albino rats weighing between 180 and 200 g were divided into 4 groups, with 8 animals in each group. The rats were housed in rooms with a temperature of 22 ± 2 °C, a 12 h light and 12 h dark cycle, and ad libitum access to food and water during the 7-day adaptation period and the 35-day experimental period. All experimental procedures were conducted in accordance with the guidelines for the care and use of laboratory animals and were approved by the Kırşehir Ahi Evran University Animal Ethics Committee (approval number: 2023/23-03).

The groups were formed as follows:

Control group (Control, n:8): Control group received tap drinking water. An equal dose of saline of hypothyroid group was injected intraperitoneally for 35 days.

Hypothyroidism group (T, n:8): 0.03% 2-mercapto-1-methyl-imidazole (methimazole)(Thyramazol, Abdi ibrahim drug, Turkey) was added to the drinking water daily to induce hypothyroidism for 35 days [21]. An equal dose of saline of hypothyroid group was injected intraperitoneally for 35 days.

Hypothyroidism + Spexin (T + Spx, n:8): 0.03% methimazole was added to the drinking water daily and intraperitoneal (ip) injection of 25 µg/kg spexin for 35 days.

Spexin (S, n:8): 25 µg/kg spexin (in saline)(Novopro bioscience, Shangai, China) was injected intraperitoneally for 35 days [22,23].

Behavioral Testing of Experimental Groups

The Open Field Test (OFT) and the Forced Swim Test (FST), two commonly used assessments, were conducted to evaluate the behavioral responses of the experimental groups. These tests assess locomotor activity, anxiety-related behavior, and depressive-like behavior to determine the impact of spexin on hypothyroidism.

### 2.2. Open Field Test

The open field test is a traditional experimental method used to evaluate general movement and anxiety-related behaviors [24]. Each animal underwent the open field test a single time. The tests were carried out during the light phase of the illumination cycle, and all experimental groups were assessed within the same timeframe. The apparatus is a square enclosure made of plexiglass (60 cm × 60 cm) with 50 cm high walls surrounding it. Prior to each animal’s test, the apparatus was sanitized with 70% ethanol. At the beginning of the test, the animals were placed in the center of the open field and given 5 min to explore freely. In this 5 min exploration, tests were conducted using a ceiling-mounted video camera in the behavioral room, which was linked to a computer for image recording. To analyze and process the behavioral parameters identified in the videos, the computer software AnyMaze Video Tracking System version 7.4 was utilized for the experimental groups. The video tracking system documented total distance traveled, distance in both the center and peripheral zones, number of entries into the center and peripheral zones parameters. Analyses were conducted blindly, and results were evaluated as mean ± standard deviation (SD).

### 2.3. Forced Swim Test

The Forced Swim Test (FST) was performed using the AnyMaze video tracking system according to the Porsolt protocol [25]. In the experiment, rats were floated in a cylindrical tank with a diameter of 30 cm containing water at a depth of 30 cm and a temperature of 23 ± 1 °C. The camera was placed on top of the tank, and behaviors were recorded and analyzed using AnyMaze 7.0 (Stoelting, Wood Dale, IL, USA) software. Animals were first subjected to a 15 min adaptation (pre-test) session, followed by a 5 min test session 24 h later. In the analysis, immobility was defined as the animal’s speed being ≤2 cm/s, and the total immobility time. Each recording was made under constant light and temperature conditions, using a single camera at 30 fps. Analyses were conducted blindly, and results were evaluated as mean ± SD.

### 2.4. Biochemical Analysis

At the end of the experiments, rats were anesthetized intraperitoneally with ketamin (50 mg/kg) and xylasine (10 mg/kg). Following anesthesia, a thoracotomy was performed, blood was collected by cardiac puncture, and the animals were euthanized. After this procedure, brain and testis tissue of the rats was quickly collected for histological analysis. The blood collected in tubes was allowed to stand upright for 40 min and then centrifuged at 3000 rpm for 10 min. After centrifugation, serum samples were collected, and the levels of TSH (BT-lab CAT.No: E0180Ra), T3 (BT-lab CAT.No: EA0042Ra), and T4 (BT-lab CAT.No: EA0027Ra) were analyzed with ELISA method (Microplate reader: BIO-TEK EL X 800-Auto strip washer: BIO TEK EL X 50). All assays were performed strictly in accordance with the manufacturer’s instructions, and absorbance values were measured using a microplate reader at 450 nm.

### 2.5. Histological Evaluation

Histological evaluation was performed at the Histology and Embryology Laboratory of Adıyaman University Faculty of Medicine. Total cortex and testis tissue samples were subjected to routine tissue tracking procedures for light microscopic analysis after a one-week post-fixation process in a 10% neutral formaldehyde solution. Routine histological processing (alcohol, xylene, and paraffin series) was performed using an automatic tissue processing device (Leica TP1020, Nussloch, Germany), and 4 µm thick sections were obtained from the prepared paraffin blocks using a Thermo Shandon Finesse ME microtome device (Thermo Fisher Scientific, Cheshire, UK) to obtain 4 µm thick sections.

### 2.6. Immunohistochemical Analysis of Irisin and KISS-1 Levels in Brain and Testicular Tissues

Immunohistochemical staining was applied to determine KISS-1 and irisin levels in both testicular and total brain cortex tissues to identify possible structural changes. Sections 4–6 µm thick were taken from paraffin blocks, placed on polylysine-coated slides, and deparaffinized. The sections were then passed through a series of graded alcohol solutions and boiled in a microwave oven (750 W) for 12 min in a citrate buffer solution at pH 6 for antigen retrieval. After boiling, the tissues were left to cool at room temperature, washed with PBS (Phosphate-Buffered Saline), and then treated with hydrogen peroxide solution for 6 min to inhibit endogenous peroxidase activity. After washing the tissues with PBS for 3 × 5 min, a blocking solution was applied for 5 min, followed by primary antibodies diluted at a 1:200 ratio (anti-kisspeptin antibody (1:200; EPR23770-189, ab275874, Abcam, London, UK), and Irisin antibody (1:200 H-067-17, Phoenix Pharmaceuticals, Inc., Burlingame, CA, USA)) were applied to the tissues, which were then incubated in a humid environment at room temperature (22–25 °C) for 60 min. After applying the primary antibody, the tissues were washed with PBS for 3 × 5 min and then incubated with a secondary antibody compatible with the primary antibody for 30 min in a humid environment at room temperature. After the secondary antibody was applied, the tissues were washed with PBS for 3 × 5 min, incubated with Streptavidin Peroxidase (TS-125-HR, Lab Vision Corporation, Fremont, CA, USA) for 30 min in a humid environment at room temperature, and then placed in PBS [26].

The 3-amino-9-ethylcarbazole (AEC) substrate + AEC chromogen solution was dropped onto the tissues, and the image signal was obtained under a light microscope. For negative control staining, the same immunohistochemical procedure was performed except that the primary antibody was omitted and replaced with PBS. Then, all groups were simultaneously washed with PBS. The tissues, which were counterstained with Mayer’s hematoxylin, were washed with PBS and distilled water and sealed with an appropriate sealing solution (Large Volume Vision Mount, TA-125-UG, Lab Vision Corporation, USA). The prepared specimens were examined and evaluated under a Leica DM500 microscope and photographed (Leica DFC295). The histoscore was created based on the prevalence (0.1: <25%, 0.4: 26–50%, 0.6: 51–75%, 0.9: 76–100%) and intensity (0: absent, +0.5: very low, +1: low, +2: moderate, +3: high) of immunoreactivity in the staining. (Histoscore = prevalence × severity) (40×).

### 2.7. Statistical Analyses

Statistical analyses were performed to evaluate serum biomarkers, immunohistochemical findings, and behavioral parameters. Data distribution was assessed using the Shapiro–Wilk test for normality. Comparisons between groups were conducted using one-way ANOVA followed by Tukey’s post hoc test for normally distributed data (biochemical and behavioral parameters). For non-normally distributed data, the Kruskal–Wallis test was applied, followed by Dunn’s post hoc test for pairwise comparisons (immunohistochemical results). Parametric data with normal distribution were expressed as mean ± standard deviation (SD), whereas non-parametric data were presented as median (minimum–maximum). Statistical significance was set at *p* < 0.05, and all analyses were conducted using Graphad Prism (ver 9.0).

## 3. Results

### 3.1. Evaluation of the Effect of Spexin on TSH, T3, and T4 in Experimental Groups

**Figure 1:**
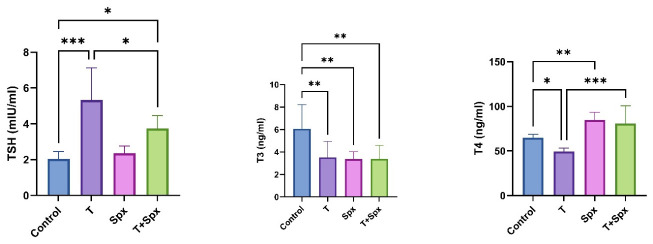
Evaluation of serum TSH, T3 and T4 levels of experimental groups. Data are represented as mean ± standard deviation (SD) (n = 8 animals/group). Control group (Control), Hypothyroidism group (T), Hypothyroidism + Spexin group (T + Spx), Spexin group (Spx). Statistical analysis was performed using one-way ANOVA followed by Tukey’s post hoc test. * *p* < 0.05, ** *p* < 0.01, *** *p* < 0.001.

In our study, TSH values were elevated in the T group (5.33 ± 1.80) compared to the control group (2.03 ± 0.41) (*p* < 0.001). TSH values of the T + Spx group (3.73 ± 0.72) were significantly lower than the T group (*p* = 0.019). Spexin administration significantly reduced TSH levels in a hypothyroid model. Spexin administration also reduced T3 levels of the Spexin group (3.37 ± 0.65) compared to the control group (6.08 ± 2.13) (*p* = 0.005). In the T,T + Spx and Spx groups (3.38 ± 1.21), spexin administration reduced T3 levels compared to the control group (3.51 ± 1.43). When examining T4 levels, a significant decrease was observed in the T group (49.41 ± 1.34) compared to the control group (64.90 ± 1.35) (*p* = 0.047). T4 levels of the T + Spx group (80.56 ± 7.10) increased significantly compared to the T group (*p* < 0.001) (Figure 1). T4 levels of the Spx group (84.79 ± 8.53) increased compared to the control group.

### 3.2. Open Field Test Parameters

In the presented study, the effects of thyroid induction and spexin administration on various behavioral parameters in the open field test were examined. These parameters include total distance traveled, time spent in the peripheral and center zones, and number of entries into the peripheral zone. Significant intergroup differences were observed in the number of entries into the outline zone (Table 1).

### 3.3. Forced Swim Test Parameters

**Figure 2:**
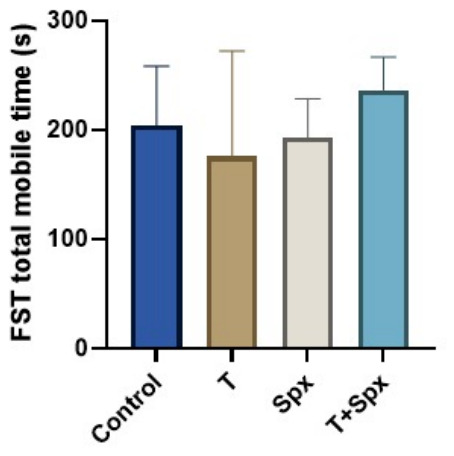
Total mobile time of experimental groups (n = 8 animals/group). Data are represented as mean ± standard deviation (SD) (n = 8 animals/group). Control group (Control), Hypothyroidism group (T), Hypothyroidism + Spexin group (T + Spx), Spexin group (Spx). Statistical analysis was performed using one-way ANOVA followed by Tukey’s post hoc test.

In the presented study, the effects of hypothyroid induction and spexin administration on various behavioral parameters in the forced swim test were examined. These parameters include total time mobile (s) and total time immobile (s). No significant differences were observed between the groups for any of these parameters. Thyroid induction or spexin administration did not induce depression-like behaviors (Figure 2).

### 3.4. Immunohistochemical Findings

#### 3.4.1. Irisin Levels in the Brain Cortex Tissue

**Figure 3:**
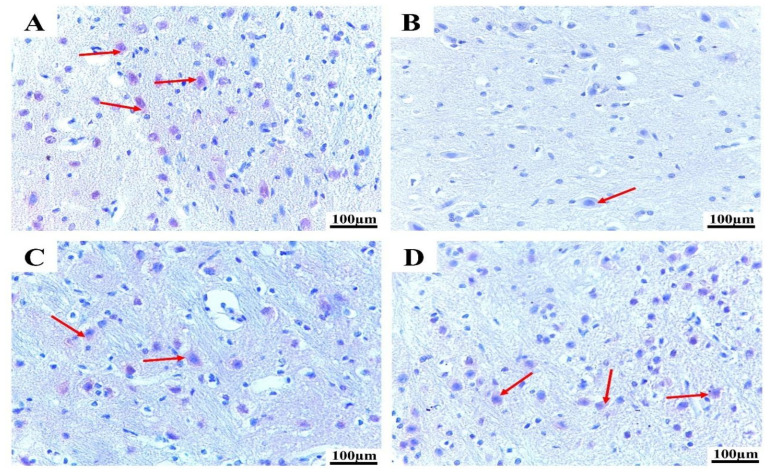
Irisin immunoreactivity in the rat brain cortex tissue was observed as light reddish-brown. Similar irisin levels were noted in the control group and the Spx group, while a decrease was observed in the T group. However, irisin levels increased in the T + Spx group following treatment. ((**A**): Control group, (**B**): T group, (**C**): T + Spx group, (**D**): Spx group; red arrow indicates irisin immunoreactivity; AEC chromogen, Mayer’s hematoxylin, scale bar: 100 µm, immunohistochemical staining).

Irisin immunoreactivity was similar in the control and Spx groups (*p* = 0.637). Compared to the control group, irisin immunoreactivity was decreased in the T group (*p* < 0.001). Furthermore, in the T + Spx group, irisin immunoreactivity in the brain cortex was increased compared to the T group (*p* < 0.05) (Figure 3 and Figure 7, Table 2).

##### KISS-1 Levels in Brain Cortex Tissue

**Figure 4:**
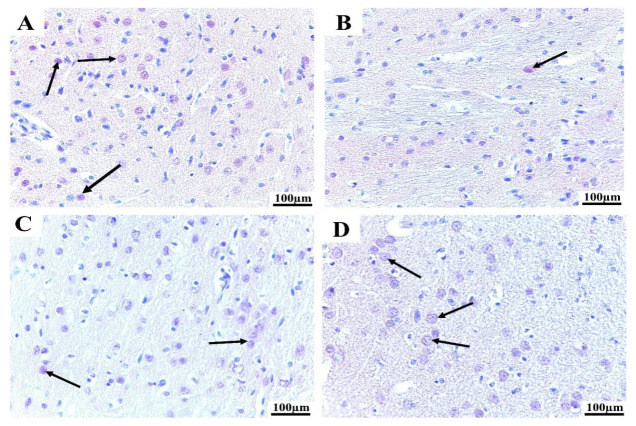
KISS-1 immunoreactivity in the rat brain cortex tissue is observed as light reddish-brown. Similar KISS-1 levels were noted in the control and Spx groups, while a decrease was observed in the T group. However, treatment with spexin increased KISS-1 levels in the T + Spx group. ((**A**): Control group, (**B**): T group, (**C**): T + Spx group, (**D**): Spx group; black arrow indicates KISS-1 immunoreactivity in the interstitial area; AEC chromogen, Mayer’s hematoxylin, scale bar: 100 µm, immunohistochemical staining).

KISS-1 immunoreactivity was similar in the control and Spx groups (*p* = 0.418). Compared to the control group, KISS-1 immunoreactivity was decreased in the T group (*p* < 0.001). Additionally, in the T + Spx group, KISS-1 immunoreactivity in the brain cortex was increased compared to the T group (*p* = 0.05) (Figure 4 and Figure 7, Table 3).

##### Irisin Levels in Testis Tissue

**Figure 5:**
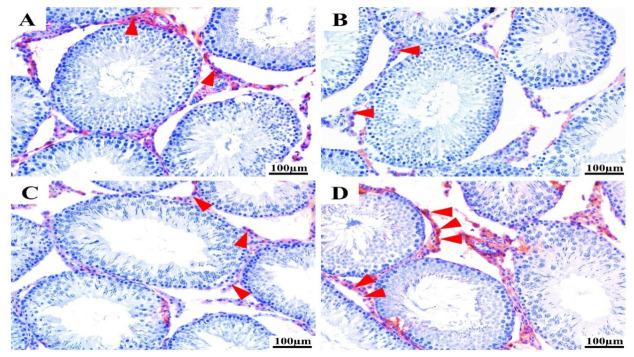
Irisin immunoreactivity in the interstitial area of rat testis tissue is observed as light reddish-brown. Similar irisin levels were noted in the control and Spx groups, while a decrease was observed in the hypothyroid group. However, treatment with spexin increased irisin levels in the T + Spx group. ((**A**): Control group, (**B**): T group, (**C**): T + Spx group, (**D**): Spx group; red arrow indicates irisin immunoreactivity in the interstitial area; AEC chromogen, Mayer’s hematoxylin, scale bar: 100 µm, immunohistochemical staining).

Irisin expression in the interstitial area of rat testis tissue was observed in all groups. Irisin immunoreactivity levels were statistically similar between the control group and the spexin group (*p* = 0.739). However, irisin immunoreactivity was significantly decreased in the T group compared to the control group (*p* < 0.05). Additionally, in the treatment group (hypothyroid + spexin), irisin immunoreactivity levels were increased compared to the hypothyroid group (*p* < 0.05) (Figure 5 and Figure 7, Table 4).

##### KISS-1 Levels in Testis Tissue

**Figure 6:**
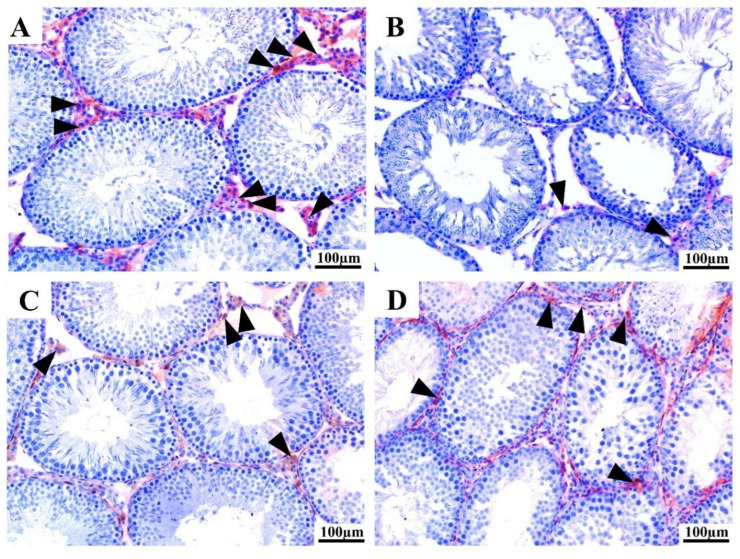
KISS-1 immunoreactivity in the interstitial area of rat testis tissue is observed as light reddish-brown. Similar KISS-1 levels were seen in the control and Spx groups, while a decrease was noted in the T group. Treatment with spexin increased KISS-1 levels in the T + Spx group. ((**A**): Control group, (**B**): T group, (**C**): T + Spx group, (**D**): Spx group; black arrow indicates KISS-1 immunoreactivity in the interstitial area; AEC chromogen, Mayer’s hematoxylin, scale bar: 100 µm, immunohistochemical staining).

**Figure 7:**
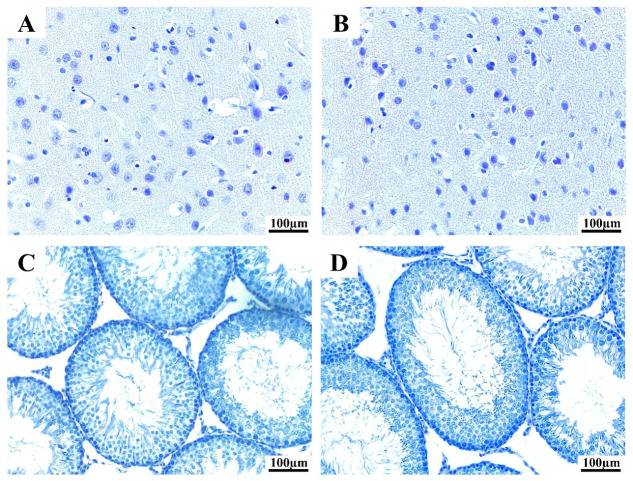
Negative control images of brain and testicular tissues obtained from immunohistochemical staining for KISS-1 and irisin ((**A**): Brain KISS-1, (**B**): Brain irisin, (**C**): Testicular KISS-1, (**D**): Testicular irisin).

KISS-1 expression was observed in the interstitial area of rat testis tissue across all groups. KISS-1 immunoreactivity levels were statistically similar between the control group and the Spx group (*p* = 0.400). However, KISS-1 immunoreactivity was significantly decreased in the T group compared to the control group (*p* < 0.05). Additionally, in the T + Spx, KISS-1 immunoreactivity levels increased compared to the T group (*p* < 0.05) (Figure 6 and Figure 7, Table 5).

## 4. Discussion

Hypothyroidism is a prevalent endocrine disorder characterized by reduced thyroid hormone production and is frequently associated with metabolic, reproductive, and neurobehavioral dysfunctions. In the present study, we investigated the effects of spexin administration on thyroid function, behavioral outcomes, and the tissue expression of irisin and KISS-1 in the cerebral cortex and testis in a methimazole-induced rat model of hypothyroidism. Our findings demonstrated that spexin treatment partially reversed hypothyroidism-associated hormonal alterations by decreasing TSH and increasing T4 levels. In addition, spexin was associated with decreased thigmotaxis, while no significant effects were observed on locomotor activity or depressive-like behavior in the Forced Swim Test. At the tissue level, hypothyroidism-induced reductions in irisin and KISS-1 expression were attenuated following spexin administration. These results suggest a potential association between spexin and neuroendocrine as well as reproductive-related pathways in the context of hypothyroidism, warranting further mechanistic investigation.

The decrease in TSH levels and increase in T4 observed in this study suggest a role of spexin on the HPG axis and are consistent with an experimental study demonstrating that central spexin infusion in healthy rats reduced TSH while increasing fT4 and fT3 levels [27]. Similarly, in obese type 2 diabetic rats, spexin injections were reported to ameliorate thyroid hypofunction as well as metabolic, oxidative, and inflammatory parameters, with these effects being mediated in part through GALR2/3 signaling [28]. While spexin successfully normalized TSH and significantly increased T4 levels, the absence of a corresponding recovery in T3 levels is an important observation. This may indicate that spexin primarily influences the hypothalamic–pituitary axis (through TSH regulation) and/or thyroidal T4 secretion, rather than peripheral deiodination processes. Alternatively, it is possible that spexin may have a modulatory effect on peripheral T4-to-T3 conversion.

In this study, the reduction in irisin and KISS-1 immunoreactivity in the brain cortex and testes under hypothyroid conditions, followed by their restoration with spexin treatment, suggests that spexin may function as a linking molecule within the metabolism–reproduction–thyroid triad. Although a study conducted in sheep reported no significant effect of spexin on the gonadotropin axis [29], these findings may reflect species-specific differences and indicate that SPX/KISS interactions in peripheral tissues (e.g., testes, adipose tissue) may be more complex. With regard to irisin, both experimental and clinical studies highlight a bidirectional and complex relationship between thyroid function and irisin levels, emphasizing that irisin may exert metabolic effects similar to those of thyroid hormones [30,31]. Moreover, the ability of irisin administration to restore fT3, fT4, and TSH levels in antidepressant-induced thyroid hormone deficiency [30], when considered together with the recovery of hypothyroidism-associated irisin reduction following spexin treatment in the present study, implies that “exercise-mimetic” peptides such as spexin and irisin may contribute to a shared compensatory mechanism in thyroid insufficiency.

Spexin is not only a metabolic regulatory peptide but also a neuropeptide involved in the regulation of mood and appetite [11]. However, the effects of exogenous spexin administration on animal behavior, particularly on parameters assessed by the Open Field Test (OFT) and Forced Swim Test (FST), remain limited in the current literature. A spexin-based galanin receptor type-2 agonist, SG2A, has been reported to play a role in anxiety- and depression-like behaviors, stress responses, and emotional memory mechanisms in corticosterone-treated mice [32]. In addition, studies conducted in methimazole-induced hypothyroid rodent models have demonstrated increased immobility in the FST, indicative of depressive-like behavior [33,34,35], whereas OFT findings have shown either unchanged locomotor activity [34] or context-dependent alterations in locomotion and anxiety-related behavior [36]. In the open field test, the tendency of animals to remain close to the walls of the apparatus, defined as thigmotaxis, is generally interpreted as an indicator of increased anxiety-like behavior [37]. In the present study, the frequency of entries into the peripheral zone was decreased in the only spexin-treated group compared to the control group. Similarly, thigmotaxis behavior was also reduced in the T + Spx group relative to controls, whereas no significant increase was observed in the T group. Despite these findings, no statistically significant differences were detected among the groups in terms of overall locomotor activity, time spent in the central zone, or FST parameters. Considering the hormonal alterations observed between groups following spexin administration, we speculate that the absence of significant behavioral differences may be associated with factors such as the administered dose and duration of treatment used in the experimental model. It is possible that behavioral effects may emerge over a longer observation period, and therefore the current experimental duration may not have been sufficient to reveal detectable behavioral alterations. In addition to, we suggest that spexin does not exhibit characteristics consistent with a potential psychostimulant agent.

Consequently, our study is able to uniquely evaluate the impact of spexin on the simultaneous expression of irisin and KISS-1 in both cortex and testicular tissues within a hypothyroid model. This allows us to evaluate the concurrent alterations in both cortical and testicular tissues, providing preliminary insights into the potential systemic shifts occurring under hypothyroid conditions. In addition to hormonal and tissue-level alterations, changes in thigmotaxis behavior were observed exclusively in the spexin-treated groups. Overall, our findings offer exploratory data on how spexin administration modulates specific tissue biomarkers in a state of thyroid deficiency, while suggesting that neurobehavioral outcomes may be altered independently of the broader and more complex endocrine-tissue interactions.

## 5. Limitations

Several limitations of this study should be acknowledged. First, the use of a single animal species and sex limits the generalizability of the findings and does not account for potential sex-specific effects of spexin. Second, receptor-level and intracellular signaling mechanisms, particularly involving GALR2/3 pathways, were not investigated, preventing definitive conclusions regarding the molecular basis of spexin’s actions. Third, the study focused on short-term outcomes; therefore, long-term efficacy and safety of spexin administration remain unknown. Additionally, standard thyroid hormone replacement (L-thyroxine) was not included as a positive control because our primary objective was to investigate the independent regulatory effects of spexin rather than comparing its efficacy to conventional therapy. Finally, no complementary quantitative validation methods, such as Western blotting or qPCR, were performed to confirm protein or gene expression levels. Immunohistochemical evaluation was based on the H-score method, which is inherently semi-quantitative. Future studies incorporating molecular, pharmacokinetic, and translational approaches are warranted.

## 6. Conclusions

In conclusion, the present study demonstrated that spexin administration significantly modulated thyroid hormone alterations in a methimazole-induced hypothyroidism model. Although spexin administration did not produce significant changes in most behavioral parameters associated with anxiety- or depression-like behaviors, it partially ameliorated hypothyroidism-related hormonal alterations and attenuated the reduction in irisin and KISS-1 expression in brain and testicular tissues. The reduction in elevated TSH levels and the concomitant increase in T4 concentrations indicate that spexin influenced the regulation of the hypothalamic–pituitary–thyroid axis under hypothyroid conditions. These findings support the emerging role of spexin as a regulatory peptide in endocrine and metabolic homeostasis.

Although the precise mechanisms through which spexin affects thyroid hormone regulation remain unclear, the observed hormonal improvements suggest a functional interaction between spexin signaling and HPT axis activity. Given the limited number of studies addressing spexin’s role in thyroid physiology, the present results contribute novel experimental evidence to this field. Future studies incorporating molecular analyses and receptor-level investigations are warranted to clarify the pathways involved and to assess whether spexin may have translational potential in the management of thyroid dysfunctions.

## Figures and Tables

**Table 1:** Open field test of experimental groups.

Behavioral Parameters of Open Field Test	Control (Mean ± SD) (n = 8)	T (Mean ± SD) (n = 8)	SPX (Mean ± SD) (n = 8)	T + Spx (Mean ± SD) (n = 8)
Distance traveled in the center line zone (m)	1.99 ± 1.17	1.78 ± 1.20	1.36 ± 0.93	1.03 ± 0.98
Distance traveled in the outline zone (s)	10.95 ± 5.88	14.13 ± 2.23	9.45 ± 3.27	10.15 ± 2.80
Total distance traveled (m)	8.10 ± 5.05	7.14 ± 5.84	6.94 ± 1.58	7.65 ± 3.97
Number of entries to the out line zone	8.66 ± 3.38 ^a^	5.50 ± 2.42	4.50 ± 1.97	3.66 ± 1.21 ^b^

^a^: Control vs. SPX *p* = 0.008 ^b^: Control vs. T + Spx *p* = 0.001 Data are represented as mean ± standard deviation (SD) (n = 8 animals/group). Control group (Control), Hypothyroidism group (T), Hypothyroidism + Spexin group (T + Spx), Spexin group (Spx). Statistical analysis was performed using one-way ANOVA followed by Tukey’s post hoc test.

**Table 2:** Irisin histoscore values in the brain cortex tissues of rats in all groups.

Experimental Groups	Irisin
Control group	0.9 (0.6–1.2)
T group	0.20 (0.20–0.40) ^a^
Spx group	0.9 (0.6–0.9)
T + Spx group	0.9 (0.2–1.2) ^b^

Data are represented as median (min-max). Control group (Control), Hypothyroidism group (T), Hypothyroidism + Spexin group (T + Spx), Spexin group (Spx). Statistical analysis was performed using Kruskal–Wallis test followed by Dunn’s post hoc test. ^a^: Control vs. T, *p* = 0.001 ^b^: T + Spx vs. T, *p* = 0.03.

**Table 3:** KISS-1 histoscore values in the rat brain cortex tissue for all groups are presented as median (min-max).

Experimental Groups	KISS-1
Control group	1.2 (0.9–1.20)
T group	0.20 (0.10–0.60) ^a^
Spx group	0.9 (0.8–1.20)
T + Spx Group	0.9 (0.6–0.90) ^b^

Data are represented as median (min-max). Control group (Control), Hypothyroidism group (T), Hypothyroidism + Spexin group (T + Spx), Spexin group (Spx). Statistical analysis was performed using Kruskal–Wallis test followed by Dunn’s post hoc test. ^a^: Control vs. T, *p* = 0.003 ^b^: T + Spx vs. T, *p* = 0.028.

**Table 4:** Irisin histoscore values in the rat testis tissue for all groups are presented as median (min-max).

Experimental Groups	Irisin
Control group	1.8 (1.2–2.7)
T group	0.80 (0.40–0.90) ^a^
Spx group	1.8 (1.2–1.8)
T + Spx group	1.2 (0.9–2.40) ^b^

Data are represented as median (min-max). Control group (Control), Hypothyroidism group (T), Hypothyroidism + Spexin group (T + Spx), Spexin group (Spx). Statistical analysis was performed using Kruskal–Wallis test followed by Dunn’s post hoc test. ^a^: Control vs. T, *p* < 0.001 ^b^: T + Spx vs. T, *p* = 0.047.

**Table 5:** KISS-1 histoscore values in the rat testis tissue for all groups are presented as median (min-max).

Experimental Groups	KISS-1
Control group	1.8 (1.2–2.4)
T Group	0.60 (0.40–0.90) ^a^
Spx group	1.8 (1.8–2.7)
T + Spx group	1.2 (0.8–2.40) ^b^

Data are represented as median (min-max). Control group (Control), Hypothyroidism group (T), Hypothyroidism + Spexin group (T + Spx), Spexin group (Spx). Statistical analysis was performed using Kruskal–Wallis test followed by Dunn’s post hoc test. ^a^: Control vs. T, *p* = 0.033 ^b^: T + Spx vs. T, *p* = 0.009.

## Data Availability

The datasets used and/or analyzed during the current study are available from the corresponding author on reasonable request.
